# Complement inhibitor therapy in atypical hemolytic uremic syndrome (aHUS): evaluating the economic impact of introducing eculizumab biosimilars in Germany

**DOI:** 10.1186/s12882-026-05118-2

**Published:** 2026-07-03

**Authors:** Isabell Kaufhold, Daniel Lewkowicz, Danila Seidel, Paul Thomas Brinkkoetter, Florian Kron

**Affiliations:** 1 VITIS Healthcare Group, Cologne, Germany; 2 Cologne Excellence Cluster on Cellular Stress Responses in Aging-Associated Diseases (CECAD), Faculty of Medicine, University of Cologne, Cologne, Germany; 3 Department I of Internal Medicine, Center for Integrated Oncology Aachen Bonn Cologne Duesseldorf (CIO ABCD) and Excellence Center for Medical Mycology (ECMM), Faculty of Medicine, University of Cologne, University Hospital Cologne, Cologne, Germany; 4 Department II of Internal Medicine, Center for Molecular Medicine Cologne, Faculty of Medicine, University Hospital of Cologne, University of Cologne, Cologne, Germany; 5 Department I of Internal Medicine, Faculty of Medicine and University Hospital Cologne, University of Cologne, Cologne, Germany; 6 Center for Integrated Oncology (CIO ABCD), Faculty of Medicine and University Hospital Cologne, University of Cologne, Cologne, Germany; 7 FOM University of Applied Sciences, Leimkugelstraße 6, 45141 Essen, Germany

**Keywords:** Atypical hemolytic uremic syndrome, Biosimilars, Cost-minimization analysis, Eculizumab, Ravulizumab

## Abstract

**Background:**

Eculizumab, a humanized monoclonal antibody, inhibits activation of complement component C5. For patients with atypical hemolytic uremic syndrome, the introduction of eculizumab has markedly improved survival, restoring life expectancy close to that of the general population. The long-acting C5 complement inhibitor (CI) ravulizumab has since expanded treatment options, offering extended dosing intervals. In 2023, the first eculizumab biosimilars received market authorization. This study aims to evaluate the short-term economic impact of CIs from a German healthcare payer perspective.

**Methods:**

An economic model with weekly cycles and a time horizon of one year was developed to assess treatment costs for eculizumab originator, eculizumab biosimilars, and ravulizumab in adult patients with atypical hemolytic uremic syndrome, based on a standard adult body weight of 77.7 kg. The analysis included direct drug acquisition costs calculated on a milligram basis, obtained from publicly available German price databases and outpatient reimbursement regulations, as well as preparation costs for infusions. Treatment costs were analyzed up to 52 weeks to reflect short-term and annual treatment periods. All costs are presented in euros (2025), and no discounting was performed.

**Results:**

Milligram-based prices were estimated at €12.92 for eculizumab biosimilar, €17.87 for eculizumab originator, and €13.63 for ravulizumab. After 13 weeks, cumulative costs were €124,941 (biosimilar), €172,466 (originator), and €127,062 (ravulizumab). After 52 weeks, costs reached €421,439, €581,836, and €352,463, respectively.

**Conclusions:**

At treatment initiation, the eculizumab biosimilar appears to be the most cost-efficient option among CIs in Germany, offering cost savings relative to both the originator and the long-acting agent. Sequential use of different CIs may therefore represent a cost-saving option.

**Supplementary Information:**

The online version contains supplementary material available at 10.1186/s12882-026-05118-2.

## Background

Atypical hemolytic-uremic syndrome (aHUS) is a rare, complement-mediated thrombotic microangiopathy (TMA) associated with high morbidity, mortality, and healthcare burden due to expensive drug treatments and hospitalizations [1, 2]. Diagnosis of aHUS requires differential diagnosis for exclusion of alternative causes of TMA, especially thrombotic thrombocytopenic purpura (TTP), shiga toxin E. coli HUS (STEC-HUS) or secondary forms of TMA related to transplantation, pregnancy, hypertension, drug exposure, autoimmune disease, malignancy or infection[3, 4]. Genetic or acquired defects in complement-regulating proteins found in 60–70% of patients, most commonly complement factors H, I, and B, or membrane cofactor protein (MCP), lead to uncontrolled activation of the alternative pathway, causing microangiopathic hemolytic anemia, thrombocytopenia, and progressive renal impairment [5–7]. Patients face a lifelong risk of multi-organ involvement, including neurological, cardiovascular, and gastrointestinal complications [5, 8].

The global annual incidence of aHUS is estimated between 0.23 and 1.9 per million population, with a prevalence at approximately 4.9 per million and with 35–42% occurring in patients under the age of 18 [1, 9]. In Germany, the CESAR study identified 142 incident aHUS patients between 2014 and 2017, corresponding in 47 patients per year and an annual incidence of 0.57 per million population, based on German total population amounting to 82,792,351 as of December 2017 [10, 11]. Extrapolated to the 2025 German SHI population, this translates into approximately 42 incident cases per year and 365 statutory health insurance (SHI) patients nationwide [12]. Despite its rarity, aHUS imposes a disproportionate economic burden due to the high annual cost of complement inhibitors and frequent hospitalizations, thus, optimizing treatment duration and improving affordability have become major clinical and policy priorities [2, 13].

Before the introduction of complement inhibitors, prognosis was poor; despite plasma exchange or infusion, up to 70% of patients progressed to end-stage renal disease or died within one year of diagnosis [5]. The introduction of eculizumab, a humanized monoclonal antibody targeting complement component C5 with a maintenance dosing interval of two weeks and approved in 2011, fundamentally changed the prognosis for aHUS [14]. In pivotal phase II and registry studies, use of eculizumab resulted into rapid control of complement-mediated hemolysis and thrombotic microangiopathy, leading to marked reductions in transfusion need and thromboembolic events and sustained improvements in renal function, survival, and quality of life [5, 8, 15–17].

Ravulizumab, a second-generation C5 inhibitor with an extended eight-week maintenance dosing interval in adult patients, demonstrated non-inferior efficacy and safety compared with eculizumab and provides per-patient cost reductions of approximately 30–35% due to less frequent administration [18–20]. However, there are recommendations suggesting that treatment duration should be individualized according to the patient’s clinical profile and disease course. The Kidney Disease: Improving Global Outcomes (KDIGO) Controversies Conference further emphasizes this point by expressing concerns about the use of long-acting complement inhibitors during the acute phase of the disease [4, 6, 21].

Recent trials demonstrated that time-limited eculizumab therapy with structured continuous monitoring can be safe and economically superior for selected patients [22]. In the Dutch CUREiHUS study, withdrawal after a median of 13 weeks and reinitiation in case of relapse may be a safe and feasible treatment strategy, reducing total medical costs to 30% of the expected lifetime expenses [13]. Similarly, the pre-specified Bayesian analysis of the UK SETS aHUS trial found no evidence that eculizumab withdrawal with structured monitoring and prompt reintroduction if necessary exposed the included patients to additional risk of harm compared with continuous treatment [23]. Orozco-Leal et al. projected potential lifetime savings of £ 4.2 million per patient for a withdrawal-and-monitoring strategy versus lifelong eculizumab dosing, with negligible loss in life years and potential quality of life gains [2]. Nevertheless, long-term follow-up data indicate that treatment discontinuation requires careful evaluation, as patients with complement gene variants or prior kidney transplantation are at increased risk of relapse and renal function decline [24, 25]. These findings underscore the need for economically sustainable treatment strategies in aHUS.

Following the expiration of Soliris^®^ patents, the first eculizumab biosimilars Epysqli^®^ (Samsung Bioepis) and Bekemv^®^ (Amgen) received European Medicines Agency (EMA) authorization in 2023, including the indication for aHUS [26, 27]. The EMA concluded biosimilarity and established therapeutic equivalence to the reference product across all approved indications [26, 27]. While the EMA establishes biosimilarity on a scientific basis, the decisions on interchangeability and substitution are made by individual EU Member States. In Germany, automatic substitution for selected biosimilars at pharmacy level was introduced in 2024 [28].

From a health-economic perspective, biosimilars play a crucial role in balancing therapeutic innovation with sustainable access to high-cost orphan therapies. By increasing price competition and reducing budgetary pressure, they enable payers to reallocate resources to other areas of unmet need and support the long-term affordability of specialized care. The introduction of biosimilar complement inhibitors offers an opportunity for greater affordability and thus access to life-saving therapy in rare diseases such as aHUS [29]. European analyses indicate that biosimilar C5 inhibitors may reduce per-patient costs by 30–50% and alleviate national expenditures substantially [29, 30]. Such savings can be reinvested to expand patient access and support innovation. Across the EU, biosimilar competition has consistently reduced list prices of biologics by 20–70%, demonstrating its system-wide impact on cost containment and equity [29, 31].

Robust quantification of the budgetary implications of biosimilar complement inhibitors is required to inform German reimbursement policy and rare-disease budget planning. This study evaluates the health-economic impact of introducing the eculizumab biosimilar Epysqli^®^ for the treatment of aHUS into the healthcare system, compared to the eculizumab originator and the long-acting follow-on ravulizumab, with a stratified one-year time horizon, reporting cumulative costs at three predefined time points (13, 26 and 52 weeks).

## Methods

The analysis aimed to estimate and compare short-term per-patient treatment costs associated with complement inhibitors for adult patients with clinically established aHUS from a German statutory health insurance perspective. A cost-minimization framework was applied, given the comparable efficacy and safety profiles of eculizumab and ravulizumab established through indirect comparisons in the absence of head-to-head trials [32, 33].

### Model structure

An economic model was developed in Microsoft Excel 365 (Version 2510, Microsoft Corporation, Redmond, WA, USA) to simulate drug acquisition cost over a one-year time horizon. Weekly cycles were selected to reflect the actual administration patterns of complement inhibitors. For each weekly cycle, treatment costs were calculated separately for each drug and subsequently aggregated to obtain cumulative treatment costs over time. To reflect time-limited, acute-phase and switching to follow-on treatment, costs were reported at 13, 26, and 52 weeks to capture short-term and annual treatment periods. All costs are expressed in 2025 euros. Due to the limited analytical time horizon, no discounting was applied. The perspective of the German SHI was applied.

### Input parameters

#### Dosing and administration

Dosing information was obtained from the official product information, published by the European Medicines Agency [14, 20, 34]. The reference adult patient weight of 77.7 kg was derived from the mean body weight of adult patients reported in German population statistics [35]. Table 1 summarizes the dosing regimens used in the model.

**Table 1:** Dosing information (dose, cycle length and cycles) for eculizumab and ravulizumab

Treatment phase	Dose (mg)	Cycle length (weeks)	Cycles
Eculizumab			
Induction	900	1	4
Maintenance	1,200	2	Ongoing
Ravulizumab			
Induction	2,700	2	1
Maintenance	3,300	8	Ongoing

Patients ≥ 40 kg. mg = milligram, kg = kilogram

#### Drug acquisition cost

As patients with aHUS are commonly treated in specialized outpatient hospital centres, acquisition costs were determined in accordance with the contractual framework for outpatient drug reimbursement at hospitals (§ 129a Sozialgesetzbuch V), and the current agreement on pricing for substances and preparations containing substances (Hilfstaxe) [36, 37]. Ex-factory price data were obtained from the publicly available German pharmacy pricing database Lauertaxe as of November 1, 2025 [38]. Drug acquisition costs for the eculizumab biosimilars, the eculizumab originator, and ravulizumab were calculated on a milligram basis. All substances are administered parenterally; therefore, pharmacy preparation costs were added for each administration. In line with German reimbursement regulations for parenteral preparations, vial wastage was not included in the base case analysis [37].

For the eculizumab biosimilar, a fixed milligram price was used [37]. For the eculizumab originator and ravulizumab, negotiated ex-factory prices were applied [37]. For all products, statutory value-added tax (19%) was added and the mandatory manufacturers rebate (7%) deducted from the ex-factory price, consistent with German SHI regulations [36–39]. A fixed pharmacy preparation fee (€ 100 per administration) was added for each drug [37]. The resulting prices per milligram are summarized in Table 2.

**Table 2:** Drug acquisition cost (mg-based) for eculizumab biosimilar, eculizumab originator and ravulizumab

	Eculizumab biosimilars (in Euro)	Eculizumab originator (in Euro)	Ravulizumab (in Euro)
Price basis	Fixed mg price	Ex-factory price	Ex-factory price
Price per mg	11.75	15.96	12.17
+ VAT tax (19%)	+ 2.23	+ 3.03	+ 2.31
- Legal manufacturer’s rebate (7%)	-1.06	-1.12	-0.85
**Price per mg for SHI**	**12.92**	**17.87**	**13.63**

VAT: value added tax, mg: milligram, SHI: statutory health

### Sensitivity analysis

To ensure the robustness and stability of the model outcomes, deterministic one-way sensitivity analyses were performed. In these analyses, the unit prices of all complement inhibitors were systematically varied by − 5%, − 10%, and − 20% relative to their respective base-case values. Each parameter was varied individually while holding all others constant to isolate its specific impact on total treatment costs. This approach aimed to capture potential price fluctuations and expected market dynamics, particularly the anticipated price erosion following the market entry and diffusion of eculizumab biosimilars. The resulting changes in cumulative per-patient treatment costs at 13, 26, and 52 weeks were recorded and compared to the base-case estimates.

## Results

Based on the model assumptions, the per-dose acquisition cost for eculizumab 900 mg (induction) and 1200 mg (maintenance) were estimated at € 11,279 and € 15,605 for the biosimilar, and € 16,184 and € 21,546 for the originator, respectively. For ravulizumab corresponding costs were € 36,902 (2,700 mg induction) and € 45,080 (3,300 mg maintenance).

Reflecting the eight-week maintenance interval of ravulizumab compared with the two-week maintenance interval of eculizumab, the cumulative number of administrations at weeks 13, 26 and 52 amounts to 9, 15 and 28 for eculizumab and 3, 4 and 8 for ravulizumab, respectively, translating into cumulative preparation costs at week 52 of approximately € 2,800 for eculizumab and € 800 for ravulizumab. At weeks 13 and 26, the corresponding cumulative preparation costs amounted to approximately € 900 and € 1,500 for eculizumab compared with € 300 and € 400 for ravulizumab.

Total treatment costs are reported in Table 3.

**Table 3:** Results total cumulated treatment costs per patient at 13, 26 and 52 weeks

Time horizon	Eculizumab biosimilar (in Euro)	Eculizumab originator (in Euro)	Ravulizumab (in Euro)
13 weeks	**124**,**941**	172,466	127,062
26 weeks	218,572	301,741	**172**,**142**
52 weeks	421,439	581,836	**352**,**463**

Bold: lowest-cost option at each time point

At 13 weeks, cumulative treatment costs per patient were € 124,941 for the eculizumab biosimilar, € 172,466 for the originator, and € 127,062 for ravulizumab (Table 3). This translates into cost savings of € 47,525 for the eculizumab biosimilar compared to its originator and € 2,121 compared to ravulizumab, respectively. Similar relative cost savings were observed at weeks 26 and 52, for eculizumab biosimilar vs. originator, amounting to € 83,168 and € 160,397 per patient, respectively. At 13 weeks, the eculizumab biosimilar yielded the lowest cumulative cost; at 26 and 52 weeks, ravulizumab represented the most cost-minimizing option, while the eculizumab originator remained consistently the highest-cost alternative. Detailed results including week-by-week costs for each complement inhibitor and cumulative totals over time are provided in Supplementary Table S1.

### Sensitivity analysis

**Fig. 1:**
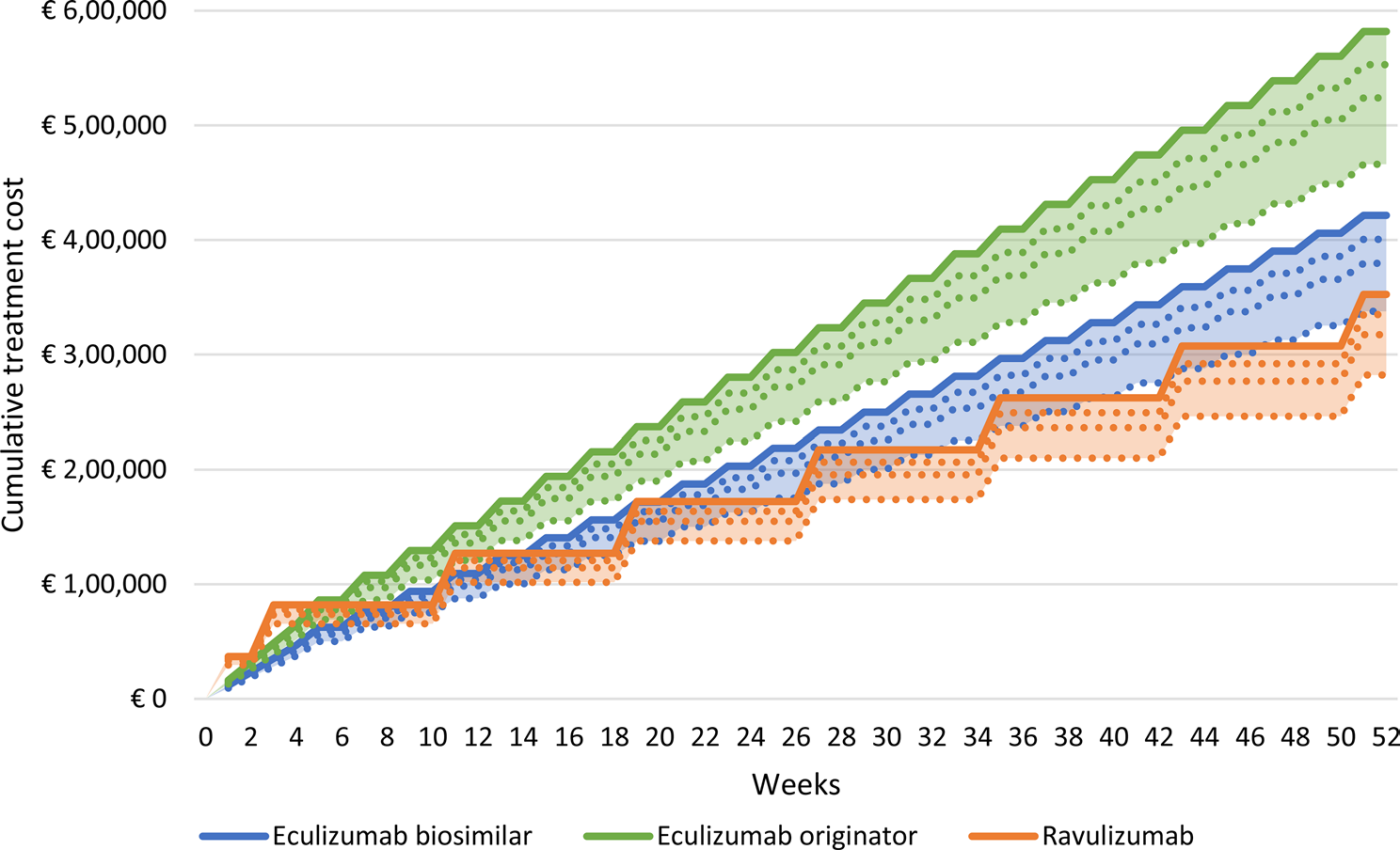
Sensitivity analysis. Solid line: base case, dotted lines from top to bottom: -5%, - 10%, -20%

The sensitivity analysis confirmed the robustness of the base-case findings, with ravulizumab becoming the lowest-cost treatment option at week 26 whereas the eculizumab originator remains the highest-cost option at all time points (Table 4; Fig. 1). At week 13, varying the acquisition price of ravulizumab by 5% led to this complement inhibitor becoming the lowest-cost treatment option overall compared to eculizumab biosimilar and originator. When varying eculizumab biosimilar, it remained the lowest-cost option until week 13, thereby widening the cost difference between itself and ravulizumab. Detailed results are provided in Supplementary Tables S2, S3 and S4.

**Table 4:** Sensitivity analysis of cumulative per-patient treatment costs of complement inhibitors for aHUS at 13, 26, and 52 weeks

Time horizon	Price variation	Eculizumab biosimilar (in Euro)	Eculizumab originator (in Euro)	Ravulizumab (in Euro)
13 weeks		**124**,**941**	172,466	127,062
	− 5%	118,739	163,888	120,724
	− 10%	112,537	155,309	114,386
	− 20%	100,133	138,153	101,710
26 weeks		218,572	301,741	**172**,**142**
	− 5%	207,718	286,729	163,555
	− 10%	196,865	271,717	154,968
	− 20%	175,158	241,693	137,794
52 weeks		421,439	581,836	**352**,**463**
	− 5%	400,507	552,884	334,880
	− 10%	379,575	523,932	317,297
	− 20%	337,711	466,028	282,130

Bold: time horizon and lowest-cost option at each time point

At week 26, cost rankings remained stable across all price variation scenarios. At week 52, the overall results also remained consistent. However, when the eculizumab biosimilar price was reduced by 20%, cumulative treatment costs fell below those of ravulizumab.

The sensitivity analysis yielded robust results for eculizumab originator, which was identified as the costliest option across all variations and time horizons.

## Discussion

This analysis estimated weekly per-patient treatment cost of complement inhibitors for the treatment of aHUS from the German statutory health insurance perspective. By applying a stratified time horizon, the study provides a differentiated assessment on cost dynamics during treatment initiation and longer-term maintenance therapy. This approach allows a nuanced understanding of economic implications in a disease where the need for rapid therapeutic action and the uncertainty of early diagnostic information play a central role.

In terms of short-term treatment over 13 weeks, eculizumab biosimilars resulted in measurable cost savings compared with both the originator and ravulizumab. The economic relevance of this early phase is amplified by the clinical challenge posed by diagnostic uncertainties due to incomplete diagnostic information during the initial days of treatment, as prompt initiation of therapy is required to achieve optimal therapeutic outcomes and mitigate the risk of enduring end-organ damage [3, 24]. This highlights that short-term treatment periods are not only of medical but also economic relevance regarding the induction phase of temporary complement inhibition, where the rapid onset of action of eculizumab and lower upfront costs of its biosimilars may offer an advantage [6, 34]. Based on the calculated incidence from CESAR study by Schönermarck et al., the potential annual cost-savings are estimated to range between € 0.09 million and € 1.99 million, if incident patients are treated with an eculizumab biosimilar during the first thirteen weeks of treatment instead of ravulizumab or the eculizumab originator, respectively. Beyond label-based dosing, additional savings may be realizable through individualized dosing strategies such as interval extension prior to discontinuation or complement-guided maintenance regimens, which have been shown to reduce cumulative complement inhibitor exposure [40].

However, these savings diminish over longer treatment horizons as the less frequent administration of ravulizumab offsets its higher price [20, 38]. For selected patients requiring continuous, long-term therapy, the extended dosing interval of ravulizumab could result into comparable or even lower overall treatment costs as well as a reduced burden on patients and caregivers due to reduced clinic visits and infusions [19, 20, 41]. The transition from eculizumab to ravulizumab, however, requires a single weight-based intravenous loading dose before the eight-week maintenance interval applies, which adds one additional infusion compared with continued eculizumab therapy [14, 27, 34]. In a scenario where all incident patients transition to long-term ravulizumab after initial treatment, annual savings of €2.90 million to €9.63 million could be realized compared with continued therapy with a biosimilar or the originator. Our findings suggest that a treatment sequence initiating with an eculizumab biosimilar for 13 weeks in cases of thrombotic microangiopathy (TMA) where atypical hemolytic uremic syndrome (aHUS) is the most probable clinical diagnosis, followed by transition to long-acting ravulizumab in patients with a sustained medical requirement for treatment, may offer an economically efficient and medically appropriate strategy within the German SHI context [42].

While the underlying methodology is transferable to other healthcare systems, the quantitative findings are specific to the German statutory health insurance context and reflect local list prices, statutory rebates and reimbursement frameworks that differ substantially across European countries. Cross-European evidence consistently shows that biosimilar competition has reduced list prices of biological medicines, and country-specific evaluations such as the Spanish cost-minimization analysis of the eculizumab biosimilar ABP 959 are broadly consistent with the qualitative direction of our findings [30, 31]. Several economic evaluations have examined treatment strategies for aHUS, yet the findings remain heterogeneous across settings [10, 19, 41, 43, 44]. Orozco-Leal et al. found that disease monitoring is cost-effective compared to lifelong eculizumab from a UK healthcare payer perspective [2]. Jang et al. assessed the regulatory pathways in Europe and development of eculizumab biosimilars and emphasized the importance of biosimilars in reducing costs and enhancing patients access [44]. In addition, Levy et al. demonstrated reduced productivity loss for ravulizumab compared with eculizumab across Germany, Italy, the UK and the US, while Wang et al. conducted a cost-minimization-analysis from a US payer perspective showing cost reductions of 32–35% with ravulizumab [19, 41]. Divergent pricing structures, reimbursement regulations, and healthcare utilization patterns across countries likely contribute to observed differences.

To the best of our knowledge, this is the first study providing economic evidence based on treatment costs for complement inhibitors in the German setting. Our results partly contrast the existing evidence, primarily due to the stratified time horizon applied in our analysis. The results from this analytical approach highlight the importance of evaluating short-term time horizons in respective diseases like aHUS, where acute or temporary treatment is highly relevant. Long-term cost assumptions may overestimate the actual economic burden.

From the perspective of the German SHI, both complement inhibitors impose substantial annual drug acquisition cost, underscoring the need for biosimilar introduction and uptake in rare diseases like aHUS. Biosimilars offer the potential to generate meaningful cost savings while maintaining equivalent clinical efficacy and safety, thereby contributing to the financial relief of the healthcare system [45]. Beyond direct cost savings, the adoption of biosimilars may generate additional reinvestment effects, as the released budget could be allocated to other innovative therapies or healthcare interventions.

Our analysis is subject to several limitations. First, the model applied a short-term time horizon of up to 52 weeks and therefore does not account for long-term treatment continuation and individual treatment patterns. Consequently, potential cost differences related to long-term efficacy, treatment persistence, or safety profiles were not considered. Second, dosing assumptions was based on the official product information and an average adult bodyweight; therefore pediatric patients, dosing adjustments and patient weight variability were not considered. Third, drug acquisition costs were derived from publicly available German drug pricing databases but are subject to confidential rebate contracts. Therefore, actual costs may vary and depend on the specific SHI. Fourth, cost-minimization analyses inherently rely on the assumption of equivalent or comparable efficacy and safety profiles between comparators; in the present analysis, this assumption was informed by indirect comparative evidence rather than direct head-to-head trials. Finally, the analysis was based on modeled assumptions and did not incorporate real-world utilization patterns or patient-level clinical outcome data.

## Conclusions

From the perspective of the German statutory health insurance, this cost-minimization analysis suggests that, for an average-weighted adult patient over a stratified time horizon of one year, eculizumab biosimilars yield substantial savings versus the originator and short-term savings versus ravulizumab during the induction phase. Overall, the results highlight the importance of biosimilar uptake to enhance cost efficiency and patient access within the German healthcare system. Future research incorporating pediatric patients, weight-based dosing, real-world utilization data and confidential pricing agreements will be valuable to confirm these findings and further inform payer decision-making. These findings underline the budget impact potential of biosimilar adoption in rare diseases such as aHUS, where high-cost therapies represent a considerable burden to the healthcare system.

## Supplementary Information

Below is the link to the electronic supplementary material.

Supplementary Material 1

## Data Availability

All data generated or analysed during this study are included in this published article and its supplementary information files.
