# Supplementary material for: Complement inhibitor therapy in atypical hemolytic uremic syndrome (aHUS): evaluating the economic impact of introducing eculizumab biosimilars in Germany
Source: BMC Nephrol. 2026 Jul 3;27:402. doi: 10.1186/s12882-026-05118-2 (PMC13340090; doi:10.1186/s12882-026-05118-2)
Supplement: Supplementary file 1 — Supplementary Material 1 [file 12882_2026_5118_MOESM1_ESM.pdf]

Supplementary Material on Complement inhibitor therapy in atypical hemolytic uremic syndrome (aHUS): Evaluating the economic impact of introducing eculizumab biosimilars in Germany by Kaufhold et al.

Table S1: Cumulated weekly treatment costs

Weeks	Eculizumab biosimilar	Eculizumab originator	Ravulizumab
1	11.729 €	16.184 €	36.902 €
2	23.458 €	32.369 €	36.902 €
3	35.187 €	48.553 €	81.982 €
4	46.915 €	64.737 €	81.982 €
5	62.521 €	86.283 €	81.982 €
6	62.521 €	86.283 €	81.982 €
7	78.126 €	107.829 €	81.982 €
8	78.126 €	107.829 €	81.982 €
9	93.731 €	129.375 €	81.982 €
10	93.731 €	129.375 €	81.982 €
11	109.336 €	150.920 €	127.062 €
12	109.336 €	150.920 €	127.062 €
13	124.941 €	172.466 €	127.062 €
14	124.941 €	172.466 €	127.062 €
15	140.546 €	194.012 €	127.062 €
16	140.546 €	194.012 €	127.062 €
17	156.151 €	215.558 €	127.062 €
18	156.151 €	215.558 €	127.062 €
19	171.757 €	237.103 €	172.142 €
20	171.757 €	237.103 €	172.142 €
21	187.362 €	258.649 €	172.142 €
22	187.362 €	258.649 €	172.142 €
23	202.967 €	280.195 €	172.142 €
24	202.967 €	280.195 €	172.142 €
25	218.572 €	301.741 €	172.142 €
26	218.572 €	301.741 €	172.142 €
27	234.177 €	323.286 €	217.223 €
28	234.177 €	323.286 €	217.223 €
29	249.782 €	344.832 €	217.223 €
30	249.782 €	344.832 €	217.223 €
31	265.387 €	366.378 €	217.223 €
32	265.387 €	366.378 €	217.223 €
33	280.993 €	387.924 €	217.223 €
34	280.993 €	387.924 €	217.223 €
35	296.598 €	409.469 €	262.303 €
36	296.598 €	409.469 €	262.303 €
37	312.203 €	431.015 €	262.303 €
38	312.203 €	431.015 €	262.303 €
39	327.808 €	452.561 €	262.303 €
40	327.808 €	452.561 €	262.303 €

41	343.413 €	474.107 €	262.303 €
42	343.413 €	474.107 €	262.303 €
43	359.018 €	495.652 €	307.383 €
44	359.018 €	495.652 €	307.383 €
45	374.623 €	517.198 €	307.383 €
46	374.623 €	517.198 €	307.383 €
47	390.229 €	538.744 €	307.383 €
48	390.229 €	538.744 €	307.383 €
49	405.834 €	560.290 €	307.383 €
50	405.834 €	560.290 €	307.383 €
51	421.439 €	581.836 €	352.463 €
52	421.439 €	581.836 €	352.463 €

Table S2: Sensitivity analysis for Eculizumab biosimilar

Weeks	Eculizumab biosimilar	- 5%	- 10%	- 20%
1	11.729 €	11.147 €	10.566 €	9.403 €
2	23.458 €	22.295 €	21.132 €	18.806 €
3	35.187 €	33.442 €	31.698 €	28.209 €
4	46.915 €	44.590 €	42.264 €	37.612 €
5	62.521 €	59.420 €	56.319 €	50.116 €
6	62.521 €	59.420 €	56.319 €	50.116 €
7	78.126 €	74.249 €	70.373 €	62.621 €
8	78.126 €	74.249 €	70.373 €	62.621 €
9	93.731 €	89.079 €	84.428 €	75.125 €
10	93.731 €	89.079 €	84.428 €	75.125 €
11	109.336 €	103.909 €	98.482 €	87.629 €
12	109.336 €	103.909 €	98.482 €	87.629 €
13	124.941 €	118.739 €	112.537 €	100.133 €
14	124.941 €	118.739 €	112.537 €	100.133 €
15	140.546 €	133.569 €	126.592 €	112.637 €
16	140.546 €	133.569 €	126.592 €	112.637 €
17	156.151 €	148.399 €	140.646 €	125.141 €
18	156.151 €	148.399 €	140.646 €	125.141 €
19	171.757 €	163.229 €	154.701 €	137.645 €
20	171.757 €	163.229 €	154.701 €	137.645 €
21	187.362 €	178.059 €	168.756 €	150.149 €
22	187.362 €	178.059 €	168.756 €	150.149 €
23	202.967 €	192.888 €	182.810 €	162.653 €
24	202.967 €	192.888 €	182.810 €	162.653 €
25	218.572 €	207.718 €	196.865 €	175.158 €
26	218.572 €	207.718 €	196.865 €	175.158 €
27	234.177 €	222.548 €	210.919 €	187.662 €
28	234.177 €	222.548 €	210.919 €	187.662 €
29	249.782 €	237.378 €	224.974 €	200.166 €
30	249.782 €	237.378 €	224.974 €	200.166 €

31	265.387 €	252.208 €	239.029 €	212.670 €
32	265.387 €	252.208 €	239.029 €	212.670 €
33	280.993 €	267.038 €	253.083 €	225.174 €
34	280.993 €	267.038 €	253.083 €	225.174 €
35	296.598 €	281.868 €	267.138 €	237.678 €
36	296.598 €	281.868 €	267.138 €	237.678 €
37	312.203 €	296.698 €	281.193 €	250.182 €
38	312.203 €	296.698 €	281.193 €	250.182 €
39	327.808 €	311.528 €	295.247 €	262.686 €
40	327.808 €	311.528 €	295.247 €	262.686 €
41	343.413 €	326.357 €	309.302 €	275.190 €
42	343.413 €	326.357 €	309.302 €	275.190 €
43	359.018 €	341.187 €	323.356 €	287.695 €
44	359.018 €	341.187 €	323.356 €	287.695 €
45	374.623 €	356.017 €	337.411 €	300.199 €
46	374.623 €	356.017 €	337.411 €	300.199 €
47	390.229 €	370.847 €	351.466 €	312.703 €
48	390.229 €	370.847 €	351.466 €	312.703 €
49	405.834 €	385.677 €	365.520 €	325.207 €
50	405.834 €	385.677 €	365.520 €	325.207 €
51	421.439 €	400.507 €	379.575 €	337.711 €
52	421.439 €	400.507 €	379.575 €	337.711 €

Table S3: Sensitivity analysis results for Eculizumab originator

Weeks	Eculizumab originator	- 5%	- 10%	- 20%
1	16.184 €	15.380 €	14.576 €	12.967 €
2	32.369 €	30.760 €	29.152 €	25.935 €
3	48.553 €	46.140 €	43.728 €	38.902 €
4	64.737 €	61.520 €	58.304 €	51.870 €
5	86.283 €	81.994 €	77.705 €	69.126 €
6	86.283 €	81.994 €	77.705 €	69.126 €
7	107.829 €	102.467 €	97.106 €	86.383 €
8	107.829 €	102.467 €	97.106 €	86.383 €
9	129.375 €	122.941 €	116.507 €	103.640 €
10	129.375 €	122.941 €	116.507 €	103.640 €
11	150.920 €	143.414 €	135.908 €	120.896 €
12	150.920 €	143.414 €	135.908 €	120.896 €
13	172.466 €	163.888 €	155.309 €	138.153 €
14	172.466 €	163.888 €	155.309 €	138.153 €
15	194.012 €	184.361 €	174.711 €	155.409 €
16	194.012 €	184.361 €	174.711 €	155.409 €
17	215.558 €	204.835 €	194.112 €	172.666 €
18	215.558 €	204.835 €	194.112 €	172.666 €
19	237.103 €	225.308 €	213.513 €	189.923 €
20	237.103 €	225.308 €	213.513 €	189.923 €

21	258.649 €	245.782 €	232.914 €	207.179 €
22	258.649 €	245.782 €	232.914 €	207.179 €
23	280.195 €	266.255 €	252.315 €	224.436 €
24	280.195 €	266.255 €	252.315 €	224.436 €
25	301.741 €	286.729 €	271.717 €	241.693 €
26	301.741 €	286.729 €	271.717 €	241.693 €
27	323.286 €	307.202 €	291.118 €	258.949 €
28	323.286 €	307.202 €	291.118 €	258.949 €
29	344.832 €	327.676 €	310.519 €	276.206 €
30	344.832 €	327.676 €	310.519 €	276.206 €
31	366.378 €	348.149 €	329.920 €	293.462 €
32	366.378 €	348.149 €	329.920 €	293.462 €
33	387.924 €	368.622 €	349.321 €	310.719 €
34	387.924 €	368.622 €	349.321 €	310.719 €
35	409.469 €	389.096 €	368.722 €	327.976 €
36	409.469 €	389.096 €	368.722 €	327.976 €
37	431.015 €	409.569 €	388.124 €	345.232 €
38	431.015 €	409.569 €	388.124 €	345.232 €
39	452.561 €	430.043 €	407.525 €	362.489 €
40	452.561 €	430.043 €	407.525 €	362.489 €
41	474.107 €	450.516 €	426.926 €	379.745 €
42	474.107 €	450.516 €	426.926 €	379.745 €
43	495.652 €	470.990 €	446.327 €	397.002 €
44	495.652 €	470.990 €	446.327 €	397.002 €
45	517.198 €	491.463 €	465.728 €	414.259 €
46	517.198 €	491.463 €	465.728 €	414.259 €
47	538.744 €	511.937 €	485.130 €	431.515 €
48	538.744 €	511.937 €	485.130 €	431.515 €
49	560.290 €	532.410 €	504.531 €	448.772 €
50	560.290 €	532.410 €	504.531 €	448.772 €
51	581.836 €	552.884 €	523.932 €	466.028 €
52	581.836 €	552.884 €	523.932 €	466.028 €

Table S4: Sensitivity analysis results for Ravulizumab

Weeks	Ravulizumab	- 5%	- 10%	- 20%
1	36.902 €	35.062 €	33.222 €	29.542 €
2	36.902 €	35.062 €	33.222 €	29.542 €
3	81.982 €	77.893 €	73.804 €	65.626 €
4	81.982 €	77.893 €	73.804 €	65.626 €
5	81.982 €	77.893 €	73.804 €	65.626 €
6	81.982 €	77.893 €	73.804 €	65.626 €
7	81.982 €	77.893 €	73.804 €	65.626 €
8	81.982 €	77.893 €	73.804 €	65.626 €
9	81.982 €	77.893 €	73.804 €	65.626 €
10	81.982 €	77.893 €	73.804 €	65.626 €
11	127.062 €	120.724 €	114.386 €	101.710 €

12	127.062 €	120.724 €	114.386 €	101.710 €
13	127.062 €	120.724 €	114.386 €	101.710 €
14	127.062 €	120.724 €	114.386 €	101.710 €
15	127.062 €	120.724 €	114.386 €	101.710 €
16	127.062 €	120.724 €	114.386 €	101.710 €
17	127.062 €	120.724 €	114.386 €	101.710 €
18	127.062 €	120.724 €	114.386 €	101.710 €
19	172.142 €	163.555 €	154.968 €	137.794 €
20	172.142 €	163.555 €	154.968 €	137.794 €
21	172.142 €	163.555 €	154.968 €	137.794 €
22	172.142 €	163.555 €	154.968 €	137.794 €
23	172.142 €	163.555 €	154.968 €	137.794 €
24	172.142 €	163.555 €	154.968 €	137.794 €
25	172.142 €	163.555 €	154.968 €	137.794 €
26	172.142 €	163.555 €	154.968 €	137.794 €
27	217.223 €	206.386 €	195.550 €	173.878 €
28	217.223 €	206.386 €	195.550 €	173.878 €
29	217.223 €	206.386 €	195.550 €	173.878 €
30	217.223 €	206.386 €	195.550 €	173.878 €
31	217.223 €	206.386 €	195.550 €	173.878 €
32	217.223 €	206.386 €	195.550 €	173.878 €
33	217.223 €	206.386 €	195.550 €	173.878 €
34	217.223 €	206.386 €	195.550 €	173.878 €
35	262.303 €	249.218 €	236.132 €	209.962 €
36	262.303 €	249.218 €	236.132 €	209.962 €
37	262.303 €	249.218 €	236.132 €	209.962 €
38	262.303 €	249.218 €	236.132 €	209.962 €
39	262.303 €	249.218 €	236.132 €	209.962 €
40	262.303 €	249.218 €	236.132 €	209.962 €
41	262.303 €	249.218 €	236.132 €	209.962 €
42	262.303 €	249.218 €	236.132 €	209.962 €
43	307.383 €	292.049 €	276.715 €	246.046 €
44	307.383 €	292.049 €	276.715 €	246.046 €
45	307.383 €	292.049 €	276.715 €	246.046 €
46	307.383 €	292.049 €	276.715 €	246.046 €
47	307.383 €	292.049 €	276.715 €	246.046 €
48	307.383 €	292.049 €	276.715 €	246.046 €
49	307.383 €	292.049 €	276.715 €	246.046 €
50	307.383 €	292.049 €	276.715 €	246.046 €
51	352.463 €	334.880 €	317.297 €	282.130 €
52	352.463 €	334.880 €	317.297 €	282.130 €
